# Analysis of HIV drug prophylaxis services cascade in healthcare workers: a cross-sectional study in China

**DOI:** 10.1186/s12879-023-08873-x

**Published:** 2024-01-02

**Authors:** Jingkun Hu, Wenting Kang, Jiahuan Guo, Jie Xu, Fan Lyu, Guang Zhang

**Affiliations:** 1 National Center for AIDS/STD Control and Prevention, Chinese Center for Disease Control and Prevention, Beijing, China; 2 Chinese Association of STD&AIDS Prevention and Control, Beijing, China

**Keywords:** Healthcare workers, HIV drug prophylaxis, Cascade, Cross-sectional study

## Abstract

**Background:**

Human immunodeficiency virus (HIV) drug prophylaxis, including post-exposure prophylaxis (PEP) and pre-exposure prophylaxis (PrEP), has not yet been generally recognized and accepted by the whole society in China, and the utilization coverage among high-risk populations is low. Healthcare workers (HCWs) are important to the promotion and implementation of HIV drug prophylaxis strategy. This study analyzed the HIV drug prophylaxis services cascade (knowledge, attitude, and service) in HCWs, and explored the correlations between PEP and PrEP.

**Methods:**

A cross-sectional study was conducted among 1066 HCWs in 20 designated hospitals for HIV antiretroviral therapy in 20 cities in China. We collected information on participants’ essential characteristics, HIV drug prophylaxis services cascade (knowledge, attitude, and service) and so on. The Chi-square test was used to analyse whether the differences and correlations between categorical variables were statistically significant, and Pearson contingency coefficient was used to analyse the strength of correlations. Multivariable logistic regression was used to analyse associated factors.

**Results:**

Among three stages of HIV drug prophylaxis services cascade, a high percentage of 1066 participants had knowledge of HIV drug prophylaxis (PEP: 78.2%, PrEP: 80.0%). Of them, almost all had supportive attitudes towards HIV drug prophylaxis (PEP: 99.6%, PrEP: 98.6%). Only about half of them would provide HIV drug prophylaxis services (PEP: 53.5%, PrEP: 48.5%). There were positive correlations between knowledge of PEP and PrEP (*r* = 0.292), between attitudes toward PEP and PrEP (*r* = 0.325), and between provision of PEP services and PrEP services (*r* = 0.555) in HCWs.

**Conclusions:**

There was a positive correlation between PEP and PrEP in HCWs. At the stage of providing HIV drug prophylaxis services, training, advocacy and education for HCWs, should be targeted and also combine PEP and PrEP to maximize the effects, so as to improve the enthusiasm of HCWs to provide HIV drug prophylaxis services.

## Introduction

The prevalence of human immunodeficiency virus (HIV) in China is low, but the HIV prevention and control remains critical, and the HIV epidemic remains one of the major public health problems in China. By the end of 2022, 1.223 million people living with HIV have been reported in China. Before 2019, the number of newly reported HIV infection and acquired immunodeficiency syndrome (AIDS) patient showed an increasing trend year by year, reaching a peak in 2019 with 151,000 cases. There were 131,000, 129,000 and 107,000 newly reported cases of HIV/AIDS in 2020, 2021 and 2022 respectively, significantly decreasing compared with the number in 2019. The newly reported cases were infected with HIV mainly through sexual transmission. In 2022, 97.6% of the newly reported cases were sexually transmitted, including 72.0% heterosexual transmission and 25.6% homosexual transmission [1]. Heterosexual transmission is complex and diverse, including commercial heterosexual sex, non-marital and non-commercial heterosexual sex, spouses and regular sexual partners. Homosexual transmission has a higher rate of infection and greater risk of transmission. This situation poses new challenges to the prevention and control of HIV in China [2, 3].

Traditional behavioral interventions such as sexual abstinence, condom promotion, fewer sexual partners, and needle exchange have significantly reduced the number of new HIV infections. However, in the situation of the HIV epidemic dominated by sexual transmission, the HIV is spreading from the high-risk population to the general population, meanwhile, the use of new social media has increased the invisibility of high-risk behaviors, so many people are still at high risk of HIV infections [3–5]. Related studies have shown that behavioral interventions alone cannot fully control the sexual transmission of HIV [6–8].

The control of new HIV infections requires a combination of sociological, behavioral and biomedical approaches [8]. In addition to the previous strategies of publicity and education, behavioral intervention, HIV counseling and testing, antiretroviral therapy and so on, HIV drug prophylaxis is important for reducing new HIV infections. Being a biomedical prevention strategy to reduce new HIV infections, HIV drug prophylaxis, including post-exposure prophylaxis (PEP) and pre-exposure prophylaxis (PrEP) shows great significance to achieve the UNAIDS goal of 95% of people at risk of HIV infection receiving combination prophylaxis and ending the epidemic by 2030 [7]. The accessibility, acceptability, effectiveness of HIV drug prophylaxis have been proven in many countries around the world and HIV drug prophylaxis has been implemented [9, 10]. The World Health Organization issued relevant guidelines and issued new implementation guidelines for a simplified and differentiated approach to PrEP service delivery in 2022, and the United States updated its guidelines for HIV drug prophylaxis in 2022 [11–13].

China has also attached great importance to the promotion and implementation of HIV drug prophylaxis [14]. In *the Implementation Plan to Control the Spread of AIDS (2019–2022)*, the Chinese government called for the exploration of promotion model of HIV drug prophylaxis. In addition, the *Technical Guidelines for HIV Post-exposure Prophylaxis (trial version)* [15] and the *Consensus Statement on HIV pre-exposure prophylaxis in China* [16] have been issued in China. Although HIV drug prophylaxis has been continuously tired to promote and implement in China, it has not yet been generally recognized and accepted by the whole society, and the utilization coverage among high-risk populations is low [17, 18].

Healthcare workers (HCWs), as the deliverer of HIV drug prophylaxis, play an important role to the promotion and implementation of HIV drug prophylaxis strategy [19]. With the knowledge of HIV drug prophylaxis and having supportive attitudes towards it, HCW’s action in providing HIV drug prophylaxis services to high-risk populations and guiding those who are eligible for HIV drug prophylaxis to use it, will contribute to the promotion and implementation of HIV drug prophylaxis strategy in China.

Previous studies on HIV drug prophylaxis in China had mainly focused on the perspective of users of the HIV drug prophylaxis [20–24], including knowledge, willingness to accept, willingness to pay, and adherence to HIV drug prophylaxis of high-risk populations such as men who have sex with men, people who have commercial sex, transgender women and so on. However, few studies on the providers of HIV drug prophylaxis service have been found, and information about understanding of knowledge, attitude, service provision, counseling, assessment and prescription of HIV drug prophylaxis among HCWs is insufferent. In *the Consensus Statement on HIV pre-exposure prophylaxis in China* [16], PrEP is recommended for people who have regularly used PEP or are willing to use PEP. However, in practice, HCWs rarely implement this recommendation. Few researchers have explored the correlations between PEP and PrEP cascade among HCWs in the past.

This study analyzes the HIV drug prophylaxis services cascade (knowledge, attitude, and service) among HCWs in 20 designated hospitals for HIV antiretroviral therapy in 20 cities in China, and explores the correlations between PEP and PrEP.

## Methods

### Participants

Participants were recruited from the Infectious Disease Departments of 20 designated hospitals for HIV antiretroviral therapy of 20 cities in China in 2022. These 20 cities were on the list of project cities of *the Fourth Round of China's Comprehensive AIDS Response (China CARES)*. These project cities have done a good job in AIDS prevention and control, and were also very experienced in the promotion and implementation of PEP and PrEP, forming an excellent example in China. These 20 cities were located in seven regions of China and these seven regions represented different economic area in China. The seven regions included East China (Fuzhou, Hangzhou, Hefei, Nanchang, Nanjing, Qingdao), Central China (Wuhan, Changsha, Zhengzhou), North China (Shijiazhuang, Taiyuan, Tianjin), Northeast China (Harbin, Changchun), South China (Nanning), Southwest China (Chengdu, Guiyang, Kunming, Chongqing), Northwest (Xi 'an). HCWs who met the following inclusion criteria were recruited for this study: (1) working in the Infectious Disease Departments of the designated hospital for HIV antiretroviral therapy; (2) having at least 3 years working experience; (3) being willing to participate in the study voluntarily.

### Measures

This study was a cross-sectional survey using a convenience sampling method. The trained investigators first contacted with the leaders of the Infection Disease Departments of the designated hospital for HIV antiretroviral therapy and obtained their consent. With their recommendation, the HCWs meeting the inclusion criteria were selected from the Infection Disease Departments by accidental sampling. After introducing the aim, significance and related things of the survey, questionnaires were issued with informed consent. The Chinese online survey tool, Wenjuanxing (www.wjx.cn), was used to administer the survey. The sample size was computed via the formula ${\text{N}}= \frac{{{{\text{Z}}}^{2}}_{1-\mathrm{\alpha }/2}\times {\text{pq}}}{{{\text{d}}}^{2}}$, where $\mathrm{\alpha }=0.05$, ${{\text{Z}}}_{1-\alpha /2}$=1.96 and q = 1-p. The estimated acceptable margin of error for proportion d = 0.15p, and the proportion of HCWs who provided HIV drug prophylaxis services was estimated at 30% in China [25]. Finally, the minimum sample size was estimated at about 934. In the process of questionnaire design, literature review on the current status of knowledge of HIV drug prophylaxis home and abroad, consulting with experts, and communicating with HCWs of the Infectious Disease Departments were applied.

The questionnaire was composed of three parts: the first part contained essential characteristics. The second part contained PEP service cascade: knowledge of PEP, attitudes toward PEP, and provision of PEP services (including providing PEP consultation or prescribing PEP drug). The third part contained PrEP service cascade: knowledge of PrEP, attitudes toward PrEP, and provision of PrEP services (including providing PrEP consultation or prescribing PrEP drug). We have conducted pre-survey in eight cities: Beijing, Shenzhen, Kunming, Chengdu, Chongqing, Changsha, Jinan and Nanjing in 2021. In the pre-survey, the reliability and validity of the questionnaire were tested. In the PEP section, Cronbach’s alpha was 0.978, the split-half reliability was 0.976, the KMO(Kaiser–Meyer–Olkin) was 0.965 and Bartlett spherical test (*P* < 0. 01). In the PrEP section, Cronbach’s alpha was 0.989, the split-half reliability was 0.990, the KMO(Kaiser–Meyer–Olkin) was 0.958 and Bartlett spherical test (*P* < 0. 01).

In order to ensure the quality of the survey, before survey, the authors trained on-site investigators. During the survey, on-site investigators guided participants to follow rules to answer questionnaires. After the survey, trained investigators carefully checked the quality of questionnaire. For the questionnaire with missing items, the participants were identified according to the questionnaire code and then to fill in missing items. For the questionnaire with errors, participants were identified according to the questionnaire code and then to make correction. If it could not be filled or corrected, the questionnaire will be deleted.

#### Knowledge of PEP

The knowledge of PEP includes eight questions.

1. What do you think PEP drug is use for? (The correct answer is "Prevention of HIV infection").
2. Which of the following situations do you think need to take PEP drug? (The correct answer is "Had HIV-prone behavior within the last 72 h").
3. Do you think it is necessary to have a negative HIV test result before taking PEP drug? (The correct answer is "Yes").
4. How long do you think a course of PEP drug should be? (The correct answer is "28 days").
5. Do you think it is necessary to take PEP drug every day during a course of treatment? (The correct answer is "Yes").
6. Do you think it is necessary to use the condom during sex while taking PEP drug? (The correct answer is "Yes").
7. Do you think it is necessary to get the HIV test after stopping PEP drug? (The correct answer is "Yes").
8. Do you think it is necessary to receive regular follow-up after taking PEP drug? (The correct answer is "Yes").

The participant is considered to have knowledge of PEP if he or she answers six or more questions correctly [25, 26].

#### Knowledge of PrEP

The knowledge of PrEP includes eight questions.

1. What do you think PrEP drug is use for? (The correct answer is "Prevention of HIV infection").
2. Which of the following people do you think need to take PrEP drug? (The correct answer is "Men who have sex with men, female sex workers and drug users who are not yet infected with HIV, but are at high risk").
3. Do you think it is necessary to have a negative HIV test result before taking PrEP drug? (The correct answer is "Yes").
4. What the correct way do you think is to take PrEP drug? (The correct answer is "Daily or On-demand").
5. Do you know what the number of pills is for each of the three doses of PrEP drug on-demand? (The correct answer is "2, 1, 1").
6. Do you think it is necessary to use the condom during sex while taking PrEP drug? (The correct answer is "Yes").
7. Do you think it is necessary to get the HIV test after stopping PrEP drug? (The correct answer is "Yes").
8. Do you think it is necessary to receive regular follow-up during or after taking PrEP drug? (The correct answer is "Yes").

The participant is considered to have knowledge of PrEP if he or she answers six or more questions correctly [25, 26].

#### Attitude toward PEP/PrEP

In the questionnaire, we asked two questions: "What is your attitude towards PEP?" and "What is your attitude towards PrEP?". By asking these two questions, we can know whether HCWs support PEP/PrEP or not.

#### Services provision of PEP/PrEP

In the questionnaire, we asked two questions: "Have you ever provided PEP services (including providing PEP consultation or prescribing PEP drug) to patients?" and "Have you ever provided PrEP services (including providing PrEP consultation or prescribing PrEP drug) to patients?". By asking these two questions, we can know whether HCWs provided PEP/PrEP services or not.

#### Level of hospital

In China, hospitals are graded according to their scale, scientific research direction, human resources and technical strength, medical hardware and equipment, etc. Hospitals are divided into Grade 3A hospitals, Grade 2A hospitals, and Grade 1A hospitals in descending order.

#### Cascade analysis

Cascade analysis is one of the many tools that now populate the ever growing field of a range of overlapping disciplines such as implementation science or operations research or health systems research. Its power is in highlighting the gaps in implementation along a particular well characterised pathway [27]. Cascade analysis is widely used in HIV care and HIV prevention [28–30]. In this paper, the HIV drug prophylaxis services cascade in HCWs consists of three stages, which are knowledge, attitude and service.

### Statistical analysis

SAS (version 9.4, SAS Institute Inc., Cary, NC, USA) was used for statistical analysis. Qualitative data was described by frequency and percentage, and quantitative data that did not conform to a normal distribution was described by Median (IQR). The Chi-square test was used to analyse whether the differences among categorical variables were statistically significant, and variables with statistically significant differences were included in multivariate Logistic regression analysis. The Chi-square test was used to analyse whether the correlations between categorical variables were statistically significant, and Pearson contingency coefficient was used to analyse the strength of correlations. *P*-value of < 0.05(two-tailed) was considered statistically significant.

## Results

A total of 1,258 questionnaires were sent to in this survey and 1,066 qualified questionnaires were finally obtained, with a response rate of 84.7%.

### Essential characteristics of participants

Among the 1,066 HCWs, 35.7% was between the ages of 30 and 39, the median age (IQR) was 36 (30–45) years old. 80.6% was female. 82.2% had bachelor's degree. 67.9% worked in general hospitals. 61.6% worked in grade 3A hospitals. The percentage of those who had worked on HIV/AIDS treatment and care was 52.2% (Table 1).

**Table 1:** Essential characteristics of 1066 healthcare workers

Essential characteristics	Frequency	Percentage
Age, years		
20–29	293	27.5
30–39	381	35.7
40–49	252	23.6
50–69	140	13.2
Gender		
Male	207	19.4
Female	859	80.6
Education		
High school/junior high school or below	31	2.9
Bachelor's degree	876	82.2
Postgraduate or above	159	14.9
Professional title		
None	86	8.1
Junior	413	38.7
Intermediate	344	32.3
Associate Senior	178	16.7
Senior	45	4.2
Type of hospital		
General Hospital	724	67.9
Infectious Hospital	342	32.1
Level of hospital		
Grade 1A or below	117	11.0
Grade 2A	292	27.4
Grade 3A	657	61.6
Whether had worked on HIV/AIDS treatment and care		
No	509	47.8
Yes	557	52.2

### PEP service cascade and PrEP service cascade

**Fig. 1:**
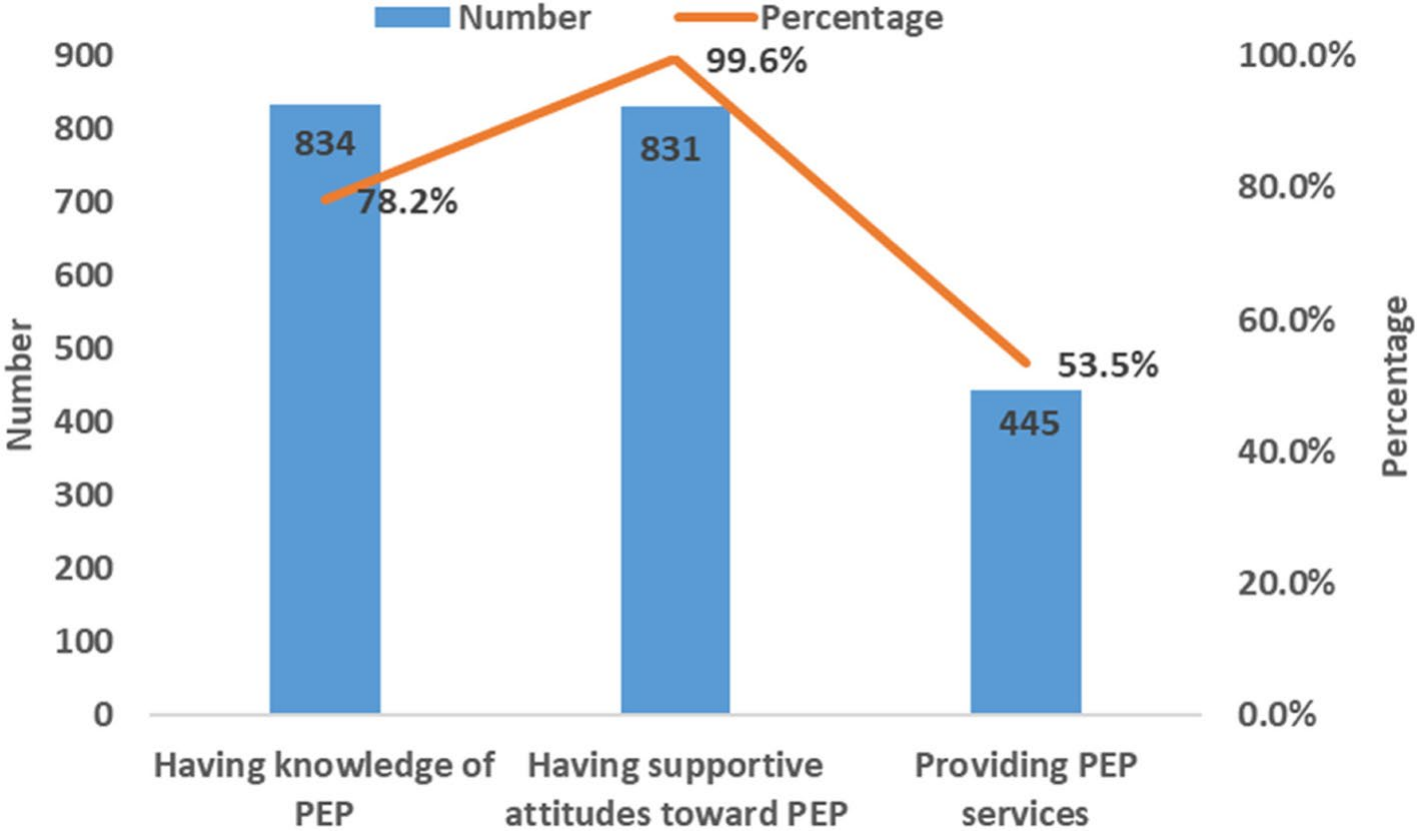
PEP service cascade in healthcare workers

Of the 1066 HCWs, 78.2% (834/1066) had knowledge of PEP. Of them, 99.6% (831/834) had supportive attitudes toward PEP. Of them, 53.5% (445/831) provided PEP services (Fig. 1).

**Fig. 2:**
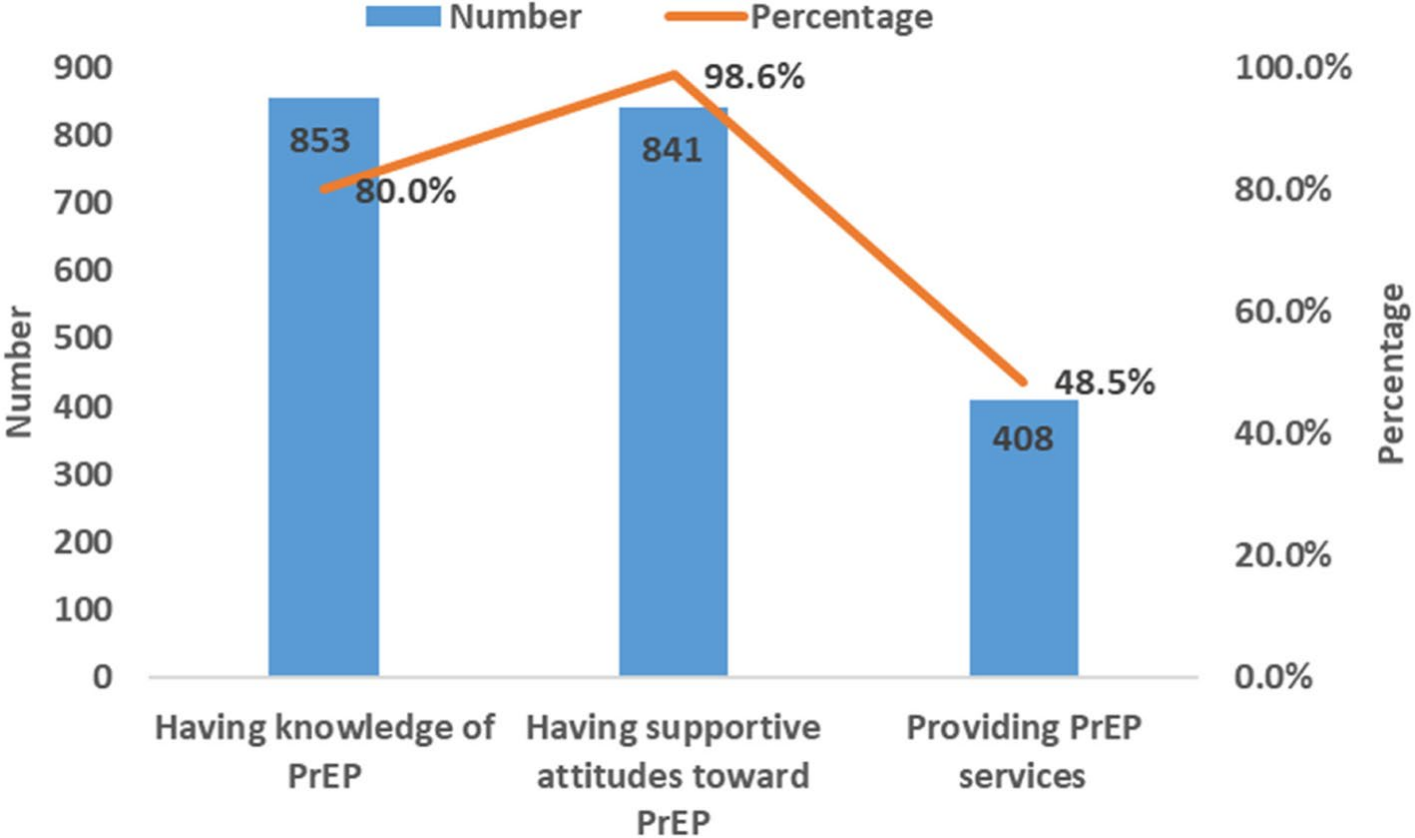
PrEP service cascade in healthcare workers

Of the 1066 HCWs, 80.0% (853/1066) had knowledge of PrEP. Of them, 98.6% (841/853) had supportive attitudes toward PrEP. Of them, 48.5% (408/841) provided PrEP services (Fig. 2).

Of the 1066 HCWs, there were statistically significant differences (*p* < 0.05) in having knowledge of PEP in terms of age, education, professional title, type of hospital, level of hospital, whether had worked on HIV/AIDS treatment and care. Professional title, type of hospital, level of hospital, whether had worked on HIV/AIDS treatment and care were the associated factors of having knowledge of PEP. Of the 834 HCWs who had knowledge of PEP, there were no statistically significant differences (*p* > 0.05) in attitudes toward PEP in terms of age, gender, education, professional title, type of hospital, level of hospital, whether had worked on HIV/AIDS treatment and care. Of the 831 HCWs who had supportive attitudes toward PEP, there were statistically significant differences (*p* < 0.05) in providing PEP services in terms of age, education, professional title, type of hospital, level of hospital, whether had worked on HIV/AIDS treatment and care. Age, type of hospital, whether had worked on HIV/AIDS treatment and care were the associated factors of providing PEP services (Tables 2 and 3).

**Table 2:** Relationship between HCWs’ essential characteristics and PEP service cascade

Essential characteristics	Participants	Having knowledge of PEP			Having supportive attitudes toward PEP		Providing PEP services		
		n (%^a^)	*X*^*2*^	*P*	n (%^b^)	*P*	n (%^c^)	*X*^*2*^	*P*
Age,years			13.105	0.004		0.508^d^		24.396	< 0.001
20–29	293	208 (71.0)			206 (99.0)		80 (38.8)		
30–39	381	313 (82.2)			312 (99.7)		178 (57.1)		
40–49	252	202 (80.2)			202 (100.0)		122 (60.4)		
50–69	140	111 (79.3)			111 (100.0)		65 (58.6)		
Gender			0.549	0.459		1.000^d^		0.061	0.805
Male	207	158 (76.3)			158 (100.0)		86 (54.4)		
Female	859	676 (78.7)			673 (99.6)		359 (53.3)		
Education			33.159	< 0.001		0.480^d^		29.798	< 0.001
High school/junior high school or below	31	16 (51.6)			16 (100.0)		4 (25.0)		
Bachelor's degree	876	671 (76.6)			669 (99.7)		335 (50.1)		
Postgraduate or above	159	147 (92.5)			146 (99.3)		106 (72.6)		
Professional title			50.953	< 0.001		0.395^d^		37.508	< 0.001
None	86	45 (52.3)			44 (97.8)		12 (27.3)		
Junior	413	311 (75.3)			310 (99.7)		150 (48.4)		
Intermediate	344	290 (84.3)			289 (99.7)		161 (55.7)		
Associate Senior	178	146 (82.0)			146 (100.0)		85 (58.2)		
Senior	45	42 (93.3)			42 (100.0)		37 (88.1)		
Type of hospital			77.690	< 0.001		0.563^d^		144.351	< 0.001
General Hospital	724	511 (70.6)			510 (99.8)		189 (37.1)		
Infectious Hospital	342	323 (94.4)			321 (99.4)		256 (79.8)		
Level of hospital			72.815	< 0.001		1.000^d^		67.274	< 0.001
Grade 1A or below	117	70 (59.8)			70 (100.0)		19 (27.1)		
Grade 2A	292	195 (66.8)			194 (99.5)		68 (35.1)		
Grade 3A	657	569 (86.6)			567 (99.7)		358 (63.1)		
Whether had worked on HIV/AIDS treatment and care			135.119	< 0.001		0.290^d^		231.121	< 0.001
No	509	320 (62.9)			320 (100.0)		65 (20.3)		
Yes	557	514 (92.3)			511 (99.4)		380 (74.4)		
Total	1066	834 (78.2)			831 (99.6)		445 (53.5)		

^a^% = number of people who have knowledge of PEP/number of participants*100%; ^b^% = number of people who have supportive attitudes toward PEP/number of people who have knowledge of PEP*100%; ^c^% = number of people who provide PEP services/number of people who have supportive attitudes toward PEP*100%; ^d^using Fisher's exact test

**Table 3:** Associated factors of having knowledge of PEP and providing PEP services in HCWs

Essential characteristics	Having knowledge of PEP		Providing PEP services	
	*aOR(95%CI)*	*P*	*aOR(95%CI)*	*P*
Age, years				
20–29	1.00		1.00	
30–39	1.00(0.64,1.57)	0.997	1.75(1.06,2.89)	0.029
40–49	0.74(0.39,1.37)	0.334	2.11(1.07,4.18)	0.031
50–69	0.75(0.35,1.60)	0.455	2.04(0.90,4.63)	0.089
Education				
High school/junior high school or below	1.00		1.00	
Bachelor's degree	1.31(0.58,2.98)	0.519	1.63(0.42,6.29)	0.482
Postgraduate or above	2.14(0.74,6.22)	0.161	2.66(0.62,11.36)	0.188
Professional title				
None	1.00		1.00	
Junior	1.80(1.06,3.06)	0.029	1.11(0.48,2.60)	0.808
Intermediate	2.97(1.52,5.80)	0.002	0.84(0.33,2.14)	0.711
Associate Senior	2.86(1.25,6.54)	0.013	0.71(0.25,2.07)	0.533
Senior	5.01(1.17,21.48)	0.030	3.99(0.89,17.88)	0.071
Type of hospital				
General Hospital	1.00		1.00	
Infectious Hospital	3.03(1.77,5.18)	< 0.001	2.95(2.02,4.32)	< 0.001
Level of hospital				
Grade 1A or below	1.00		1.00	
Grade 2A	1.46(0.90,2.37)	0.121	1.88(0.95,3.71)	0.068
Grade 3A	2.02(1.24,3.30)	0.005	1.70(0.89,3.23)	0.108
Whether had worked on HIV/AIDS treatment and care				
No	1.00		1.00	
Yes	3.55(2.38,5.29)	< 0.001	6.78(4.58,10.02)	< 0.001

Of the 1066 HCWs, there were statistically significant differences (*p* < 0.05) in having knowledge of PrEP in terms of education, professional title, type of hospital, level of hospital, whether had worked on HIV/AIDS treatment and care. Type of hospital, whether had worked on HIV/AIDS treatment and care were the associated factors of having knowledge of PrEP. Of the 853 HCWs who had knowledge of PrEP, there were no statistically significant differences (*p* > 0.05) in attitudes toward PrEP in terms of age, gender, education, professional title, type of hospital, level of hospital, whether had worked on HIV/AIDS treatment and care. Of the 841 HCWs who had supportive attitudes toward PrEP, there were statistically significant differences (*p* < 0.05) in providing PrEP services in terms of age, education, professional title, type of hospital, level of hospital, whether had worked on HIV/AIDS treatment and care. Professional title, type of hospital, level of hospital, whether had worked on HIV/AIDS treatment and care were the associated factors of providing PrEP services (Tables 4 and 5).

**Table 4:** Relationship between HCWs’ essential characteristics and PrEP services cascade

Essential characteristics	Participants	Having knowledge of PrEP			Having supportive attitudes toward PrEP		Providing PrEP services		
		n (%^a^)	*X*^*2*^	*P*	n (%^b^)	*P*	n (%^c^)	*X*^*2*^	*P*
Age,years			2.400	0.494		0.516^d^		26.176	< 0.001
20–29	293	229 (78.2)			225 (98.3)		78 (34.7)		
30–39	381	312 (81.9)			308 (98.7)		156 (50.7)		
40–49	252	197 (78.2)			193 (98.0)		112 (58.0)		
50–69	140	115 (82.1)			115 (100.0)		62 (53.9)		
Gender			1.653	0.199		0.252^d^		0.731	0.393
Male	207	159 (76.8)			155 (97.5)		80 (51.6)		
Female	859	694 (80.8)			686 (98.9)		328 (47.8)		
Education			11.539	0.003		0.073^d^		13.811	0.001
High school/junior high school or below	31	19 (61.3)			19 (100.0)		6 (31.6)		
Bachelor's degree	876	696 (79.5)			689 (99.0)		319 (46.3)		
Postgraduate or above	159	138 (86.8)			133 (96.4)		83 (62.4)		
Professional title			16.504	0.002		0.155^d^		41.886	< 0.001
None	86	55 (64.0)			53 (96.4)		14 (26.4)		
Junior	413	332 (80.4)			330 (99.4)		141 (42.7)		
Intermediate	344	279 (81.1)			275 (98.6)		138 (50.2)		
Associate Senior	178	148 (83.2)			144 (97.3)		81 (56.3)		
Senior	45	39 (86.7)			39 (100.0)		34 (87.2)		
Type of hospital			62.910	< 0.001		0.384^d^		74.770	< 0.001
General Hospital	724	531 (73.3)			525 (98.9)		194 (37.0)		
Infectious Hospital	342	322 (94.2)			316 (98.1)		214 (67.7)		
Level of hospital			27.291	< 0.001		0.590^d^		52.652	< 0.001
Grade 1A or below	117	80 (68.4)			80 (100.0)		18 (22.5)		
Grade 2A	292	215 (73.6)			213 (99.1)		76 (35.7)		
Grade 3A	657	558 (84.9)			548 (98.2)		314 (57.3)		
Whether had worked on HIV/AIDS treatment and care			79.917	< 0.001		0.137^d^		139.726	< 0.001
No	509	349 (68.6)			347 (99.4)		84 (24.2)		
Yes	557	504 (90.5)			494 (98.0)		324 (65.6)		
Total	1066	853 (80.0)			841 (98.6)		408 (48.5)		

^a^% = number of people who have knowledge of PrEP/number of participants*100%; ^b^% = number of people who have supportive attitudes toward PrEP/number of people who have knowledge of PrEP*100%;^c^% = number of people who provide PrEP services/number of people who have supportive attitudes toward PrEP*100%; ^d^using Fisher's exact test

**Table 5:** Associated factors of having knowledge of PrEP and providing PrEP services in HCWs

Essential characteristics	Having knowledge of PrEP		Providing PrEP services	
	*aOR(95%CI)*	*P*	*aOR(95%CI)*	*P*
Age,years				
20–29	–	–	1.00	
30–39	–	–	1.49(0.95,2.36)	0.085
40–49	–	–	1.80(0.98,3.30)	0.059
50–69	–	–	1.35(0.65,2.82)	0.423
Education				
High school/junior high school or below	1.00		1.00	
Bachelor's degree	1.40(0.63–3.09)	0.409	0.88(0.30,2.59)	0.811
Postgraduate or above	1.41(0.54–3.66)	0.483	0.84(0.26,2.75)	0.778
Professional title				
None	1.00		1.00	
Junior	1.64(0.97–2.80)	0.068	1.44(0.70,2.97)	0.325
Intermediate	1.44(0.83–2.50)	0.201	1.16(0.51,2.64)	0.719
Associate Senior	1.61(0.85–3.01)	0.141	1.59(0.62,4.05)	0.333
Senior	1.34(0.47–3.79)	0.588	5.15(1.34,19.81)	0.017
Type of hospital				
General Hospital	1.00		1.00	
Infectious Hospital	3.32(1.97,5.61)	< 0.001	1.67(1.17,2.38)	0.005
Level of hospital				
Grade 1A or below	1.00		1.00	
Grade 2A	1.40(0.86,2.29)	0.180	2.21(1.17,4.16)	0.015
Grade 3A	1.45(0.88,2.38)	0.141	2.32(1.26,1.26)	0.007
Whether had worked on HIV/AIDS treatment and care				
No	1.00		1.00	
Yes	2.62(1.78,3.85)	< 0.001	3.89(2.69,5.61)	< 0.001

“–” meant that no associated factors analysis was done

## Correlation between PEP and PrEP in HCWs

There was a positive correlation between knowledge of PEP and PrEP in HCWs, and pearson contingency coefficients was 0.292. There was a positive correlation between attitudes toward PEP and PrEP in HCWs, and pearson contingency coefficients was 0.325. There was a positive correlation between provision of PEP service and PEP service in HCWs, and pearson contingency coefficients was 0.555 (Table 6).

**Table 6:** Correlation between PEP and PrEP in HCWs

Variables	Knowledge of PEP	Attitudes toward PEP	Provision of PEP services
Knowledge of PrEP	*r* = 0.292^a^	–	–
Attitudes toward PrEP	–	*r* = 0.325^b^	–
Provision of PrEP services	–	–	*r* = 0.555^c^

^a^The correlation was statistically significant (*X*^*2*^ = 99.157, *p* < 0.001), r was the value of Pearson contingency coefficient; ^b^The correlation was statistically significant (*X*^*2*^ = 91.875, *p* < 0.001), r was the value of Pearson contingency coefficient;^c^The correlation was statistically significant (*X*^*2*^ = 340.269, *p* < 0.001), r was the value of Pearson contingency coefficient; “–” meant that no correlation analysis was done

## Discussion

HIV drug prophylaxis services cascade in HCWs generally consists of 3 stages (knowledge, attitude, and service). The percentage of having knowledge of HIV drug prophylaxis was high among 1066 HCWs surveyed in this study (PEP: 78.2%, PrEP: 80.0%), and they were higher compared with the results of previous studies [25, 31, 32]. This may be related to the promotion and implementation of HIV drug prophylaxis in China in recent years. Of the HCWs who had knowledge of HIV drug prophylaxis, almost all of them had supportive attitudes towards HIV drug prophylaxis (PEP: 99.6%, PrEP: 98.6%). It indicates that HCWs approve of the role of HIV drug prophylaxis in the prevention and control of HIV epidemic after having a comprehensive understanding of HIV drug prophylaxis. Of the HCWs who had supportive attitudes towards HIV drug prophylaxis, only about half of them would provide HIV drug prophylaxis services (PEP: 53.5%, PrEP: 48.5%). This reflected that most of the HCWs had supportive attitudes toward HIV drug prophylaxis, but rarely provided HIV drug prophylaxis services in practice, which was consistent with the results of related studies in the United States [33, 34]. This may be caused by HCWs' concerns about prescribing HIV drug prophylaxis to healthy people as well as concerns about drug safety and efficacy, poor adherence of drug, lack of confidence in HIV drug prophylaxis, and perceived high drug costs [35–38].

According to the results of the multivariable logistic regression, among the 1066 HCWs surveyed in this study, HCWs who had higher professional titles, worked in infectious hospitals, worked in grade 3A hospitals and had worked on HIV/AIDS treatment and care had the higher proporation of having knowledge of PEP. HCWs who had worked in infectious hospitals, and had worked on HIV/AIDS treatment and care had the higher proporation of having knowledge of PrEP. Of the HCWs who had knowledge of PEP and PrEP, the percentages of those who had supportive attitudes toward PEP and PrEP were high (nearly 100%), so there was no difference in the essential characteristics of HCWs who supported PEP and PrEP. Of the HCWs who had supportive attitudes toward PEP and PrEP, HCWs who had worked in infectious hospitals, and had worked on HIV/AIDS treatment and care were more willing to provide PEP services. HCWs who had senior title, worked in infectious hospitals, worked in grade 2A or 3A hospitals, and had worked on HIV/AIDS treatment and care were more willing to provide PrEP services. This may because these HCWs are more experienced, more knowledgeable, and more familiar with HIV-risk populations [39]. In the future, training, advocacy and education related to HIV drug prophylaxis can be targeted to HCWs with lower professional titles, working in general hospitals, working in grade 1A or 2A hospitals, so as to improve their motivation to provide PEP and PrEP services.

There showed positive correlations between PEP and PrEP among HCWs. In fact, having the knowledge of PEP helped having the knowledge of PrEP, supporting PEP facilitated supporting PrEP, and providing PEP services promoted to providing PrEP services. So, during the training, advocacy and education for HCWs, PEP and PrEP topics should be integrated, so as to maximize the effects. The correlation strength between provision of PEP and PrEP services was greater than the correlation strength between knowledge of PEP and PrEP and attitudes toward PEP and PrEP. This indicates that the influence of practice such as providing services is greater than the influence of knowledge and attitude.

There were some limitations in this study. Firstly, convenience sampling lead to some bias in the sample representativeness. Secondly, this study only quantified the provision of HIV drug prophylaxis by HCWs, but did not further analysed factors influencing their provision of PEP and PrEP services. This could be studied in the future to make up for the shortcomings of this study. Thirdly, this study did not collect information on sources of HIV drug prophylaxis knowledge obtained by HCWs, so it could not deeply explore how HCWs know about HIV drug prophylaxis, the result may help to plan knowledge dissemination in future.

## Conclusions

Among three stages of HIV drug prophylaxis services cascade in HCWs, the proportion of having knowledge of HIV drug prophylaxis and having supportive attitudes toward HIV drug prophylaxis were bigger, but the proportion of providing HIV drug prophylaxis services was smaller. There was a positive correlation between PEP and PrEP in HCWs. At the stage of providing HIV drug prophylaxis services, during the training, advocacy and education for HCWs, PEP and PrEP topics should be integrated to maximize the effects, so as to improve the enthusiasm of HCWs to provide HIV drug prophylaxis services.

## Data Availability

The datasets used and/or analyzed during the current study are not publicly available due to protect the privacy and confidentiality of participants in this study but are available upon reasonable request to the corresponding author.

## Abbreviations

HIV: Human immunodeficiency virus
AIDS: Acquired immunodeficiency syndrome
PEP: Post-exposure prophylaxis
PrEP: Pre-exposure prophylaxis
UNAIDS: The Joint United Nations Program on HIV/AIDS
HCWs: Healthcare workers
